# An in vitro study showing the three-dimensional microenvironment influence over the behavior of head and neck squamous cell carcinoma

**DOI:** 10.4317/medoral.17538

**Published:** 2011-12-06

**Authors:** Fernanda Salgueiredo-Giudice, Aline Corrêa-Abrahão, Felipe Fornias-Sperandio, Aluana M- da-Costa-Dal-Vechio, Décio dos-Santos-Pinto-Junior

**Affiliations:** 1 ………; 2 ……..; 3 ….

## Abstract

Objectives: The Head and Neck Squamous Cell Carcinoma (HNSCC) ranks sixth worldwide. The mechanisms of growth, invasion and metastasis of this pathology are extensively studied and generally related to specific variations in signaling pathways like the PI3K-Akt; however most of these competent studies have been performed bidimensionally, which may hide important questions. This study sought to analyze the influence of the microenvironment upon the behavior of HNSCC. Study Design: The status of pAkt, NF-κB and Cyclin D1 proteins was accessed through immunofluorescence and western blot methods in HNSCC cell lines originating from tongue, pharynx and metastatic lymph node when submitted to a three-dimensional culture model utilizing a matrix system. A bidimensional culture model (monolayer) was used as control. Results: The HNSCC cell lines cultured three-dimensionally exhibited a growth pattern characterized by small isolated islands, different from the control group. When the three-dimensional model was applied, two of the studied cell lines showed the same expression pattern as the bidimensional model regarding nuclear or cytoplasmatic localization, as well as reduction of all protein levels; however, the cell line originated from tongue, which specially has the epidermal growth factor receptor constitutively activated, demonstrated nuclear translocation of pAkt and also an increase in the levels of Cyclin D1. Conclusions: The results suggest the influence of the microenvironment upon the behavior of HNSCC cells due to the changed expression of proteins related to tumor growth and cellular invasion. Furthermore, intrinsically genetic conditions also played important roles over the cells, despite the culture model employed.

** Key words:**Carcinoma, squamous cell, head and neck neoplasms, extracellular matrix, cell culture techniques, signal transduction.

## Introduction

As one of the most common cancers, Head and Neck Squamous Cell Carcinoma (HNSCC) is an aggressive malignancy that arises in the oral cavity, larynx and pharynx, resulting in 500.000 new cases per year in the world (1) and nearly 11.000 deaths in the United States alone (2).

In spite of a tendency in the early detection of HNSCC (3) and advances in cancer prevention or treatment, the five-year survival rate of a patient that receives the diagnosis of HNSCC remains low (approximately 50%), which is even poorer than other cancers, such as colorectal, cervix and breast (2). Meanwhile, besides many of the most common risk factors involved in HNSCC development, such as alcohol and tobacco being well recognized, the complete knowledge of the mechanisms underlying the malignant progression of HNSCCs remains unclear (4).

In that way, many in vitro studies that focus on linking several signaling pathways with the cancer development and progression have been performed. Among the most studied proteins related to HNSCC invasion, cell proliferation and metastasis, pAkt, NF-κB and Cyclin D1 can be cited (5). Nonetheless, most of the studies account for bidimensional models over plastic surfaces and, as cancer cells reside in vivo in a three-dimensional microenvironment, the monolayer model can hide important results (6,7).

The PI3K-Akt signaling pathway, when activated, may prevent the cell death by inactivating pro-apoptotic factors and by activating transcription factors that up-regulate anti-apoptotic genes, including the transcription factor NF-κB, which is an essential component of intracellular regulatory circuitries of cell proliferation and survival (8).

In HNSCCs, the expression and activity of NF-κB is often up-regulated, and its levels increase gradually from pre-malignant lesions to invasive cancer (9,10), which suggests that NF-κB signaling pathway may play an important role at the early stages of carcinogenesis. Additionally, the crosstalk between NF-κB and STAT (signal transducer and activator of transcription) proteins leads to activation of growth promoting genes expression, such as c-myc and Cyclin D1 (11). Nevertheless, what leads to the persistent activation of NF-κB in HNSCCs is still unclear.

The cell behavior is easily modulated by the extracellular microenvironment, which is exemplified by the highly variable changes in gene expression and, subsequently, phenotype of the cancer cells cultured in different conditions (12). Thus, this work sought to evaluate the expression of pAkt, NF-κB and Cyclin D1 in HNSCC cell lines cultured with the traditional bidimensional method (monolayer) and with the three-dimensional model. Recombinant extracellular matrix composed by some components of human extracellular matrix, such as laminin, type IV collagen, nidogen, entactin and heparan sulfate (13) was used to mimic three-dimensional microenvironment.

## Material and Methods

All experiments were performed in triplicate.

Cell Lines and Culture Conditions

Three HNSCC cell lines were used [HN6 (base of tongue), HN30 (pharynx), HN31 (lymphnode)] (14) and cultured in DMEM (Dulbelco’s Modified Eagle’s Medium) supplemented with 10% fetal bovine serum, 100 U/mL penicillin and 100 µg/mL of streptomycin (Sigma-Aldrich, St. Louis, MO, USA) at 37ºC in a humidified atmosphere and 5% CO2.

Immunofluorescence Assay

The bidimensional experimental model consisted on seeding the HNSCC (HN6, HN30 and HN31) cell lines (2 x 105) over coverslips for 72 hours; the three-dimensional model was obtained exactly as the first experimental group, but the cells were seeded with 1:3 Matrigel (BD Matrigel™ Matrix, BD, Franklin Lakes, NJ, USA – product number 356231). The medium was discarded and the cells were then rinsed with phosphate buffer solution (1X PBS), fixed in cooled absolute methanol (6 minutes, –20°C), washed five times with 1X PBS and then blocked with 1% bovine serum albumin [(BSA) Sigma-Aldrich, St. Louis, MO, USA] for 30 minutes in a humidified chamber. Coverslips were incubated with anti-p-Akt1/2/3 (Ser 473) (1:50, Santa Cruz Biotechnology, Inc., Santa Cruz, CA, USA - sc7985-R), anti-NF-κB (1:50, Zymed, Invitrogen Corporation, Carlsbad, CA, USA - P65C), anti-Cyclin D1 (1:100, Santa Cruz Biotechnology, Inc., Santa Cruz, CA, USA - sc8396) diluted in blocking buffer for 90 minutes in a humidified chamber, washed five times with 1X PBS and incubated with a FITC conjugated antibody (Vector Laboratories, Ind., Burlingame, CA, USA) for 45 minutes in a dark humidified chamber. After washing five times with 1X PBS, the coverslips were mounted with mounting medium (Vectashield: DAPI, Vector Laboratories, Ind., Burlingame, CA, USA) and analyzed by fluorescence microscope (Axio Imager.A1 - Carl Zeiss).

Western blot Assay

For the bidimensional culture mode, the HNSCC cell lines (16) were seeded on 58cm2 dishes and maintained at 37ºC in a humidified atmosphere and 5% CO2 for 72 hours, and washed three times with cold 1X PBS. Cells were lysed with lysis buffer at 4°C for 20 minutes. The cells were then scraped and the lysate was collected in a microfuge tube. The lysate was cleared by centrifugation at 13.000 rpm for 20 minutes at 4ºC and the supernatant (total cell lysate) was collected, being the protein concentra-tion of all samples determined by using

the BCA method (Pierce Biotechnology, Rockford, IL, USA).

For the three-dimensional culture mode, the HNSCC cell lines (16) were re-suspended in 1:3 Matrigel and maintained at 37ºC in a humidified atmosphere and 5% CO2 for 72 hours. Later, 1X PBS was added and the sample was cleared by centrifugation at 4ºC and the supernatant (Matrigel with 1X PBS) was discarded. The cell pellet was incubated in ice-cold lysis buffer [50 mmol/L Tris–HCl (pH7.4), 1 mmol/L EDTA, 150 mmol/L NaCl, 1% Triton X-100, 1% DOC, 0.1% SDS, with freshly added protease inhibitor cocktail (Chemicon, Sigma, St. Louis, MO, USA)] for 20 minutes at 4°C and clarified by centrifugation (13.000 rpm for 20 minutes at 4ºC). Supernatant (total cell lysate) was collected and the protein concentration of all samples was also determined using the BCA method (Pierce Biotechnology, Rockford, IL, USA).

For western blotting analysis, 7μg of the protein from whole cell lysates was loaded onto each lane for gel electrophoresis and transferred to a PVDF membrane. Immunoblotting was performed using 0.1 M Tris (pH 7.5), 0.9% NaCl, 0.05% Tween- 20 with 5% nonfat dry milk or BSA (Sigma-Aldrich, St. Louis, MO, USA) as a blocking and antibody-dilution buffer, and working anti-sera for anti-p-Akt1/2/3 (Ser 473) (1:150, Santa Cruz Biotechnology, Inc., Santa Cruz, CA, USA - sc7985), anti-NF-κB (1:100, Zymed, Invitrogen Corporation, Carlsbad, CA, USA - P65C) and anti-Cyclin D1 (1:100, Santa Cruz Biotechnology, Inc., Santa Cruz, CA, USA - sc8396), followed by anti-mouse secondary antibody (IgG-HRP, Santa Cruz Biotechnology, Inc., Santa Cruz, CA, USA). Bound antibody was detected by a colorimetric method using an Opti 4CN kit (Bio-rad Laboratories, Hercules, CA, USA). Beta-actin (1:6000, Sigma-Aldrich, St. Louis, MO, USA) was used to control the total volume of each sample.

The band intensity quantification was performed using ImageJ 1.39u software (ImageJ, U. S. National Institutes of Health, Bethesda, Maryland, USA).

The statistical analysis was carried out using SPSS 11.0 (SPSS Inc., Chicago, IL, USA). Student’s t test (independent sample) was performed to compare the groups with a level of significance of 5%.

## Results

HNSCC cell lines growth patterns revealed by the immunofluorescence assay

**Figure 1:**
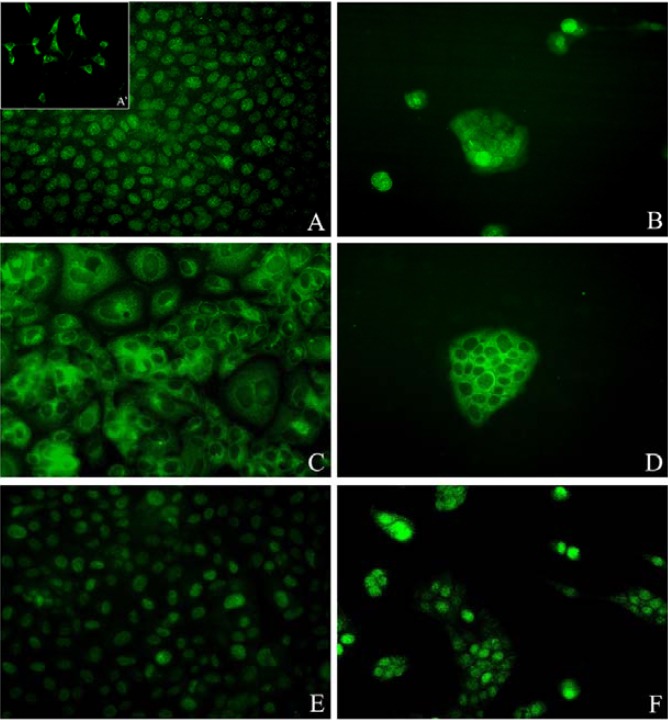
A. Nuclear pAkt expression in the HN6 cell line. A-Focal cytoplasmatic staining of pAkt in the HN6 cells; B -Nuclear translocation of pAkt in the HN6 cells when cultured three-dimensionally; C-HN30 cell line showing cytoplasmatic expression of NF-κB; D - NF-κB expression remains in the cytoplasm of HN30 cells cultured with the matrix system; E – HN31 cell line showing nuclear expression of Cyclin D1; F – Cyclin D1 expression remains in the nuclei of the HN31 cells when cultured with the three-dimensional mode.

The HNSCC cells cultured with the three-dimensional mode exhibited a growth pattern characterized by small isolated islands (Fig. 1). The other experimental group though, showed an homogenous, sheet-like, growth appearance (Fig. 1).

The three-dimensional model suggests pAkt’s nuclear translocation.

The HN6 cell line, when cultured with the bidimensional mode, presented the pAkt expression mainly in the nuclei (Fig. 1) and focally in the cytoplasm of the respective cells (Fig. 1). The expression was only nuclear when the three-dimensional culture mode was employed (Fig. 1).

The pAkt expression was cytoplasmatic for the HN30 cell line when these cells were cultured with both the experimental methods.

The HN31 cell line demonstrated nuclear expression of pAkt when cultured bidimensionally, and the expression remained in the nuclei when the three-dimensional method was employed.

NF-kB and Cyclin D1 cellular localizations remain equal for both culture methods.

The expression of these proteins was maintained in both of the experimental groups (bidimensional and three-dimensional culture modes), remaining the NF-kB staining in the cytoplasm (Fig. 1) and the Cyclin D1 staining in the nuclei (Fig. 1) of all HNSCC cell lines.

Western blotting confirms the influence of the microenvironment over HNSCC cells.

**Figure 2:**
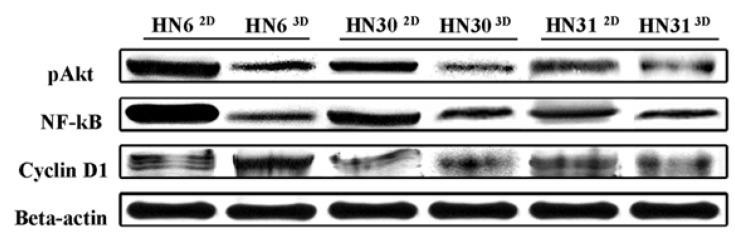
Western blotting of pAkt, NF-κB and Cyclin D1 in the respective HNSCC cell lines, indicating protein expression levels. Beta-actin was used as control of the total volume of each sample. 2D – Bidimensional model; 3D – Three-dimensional model.

**Figure 3:**
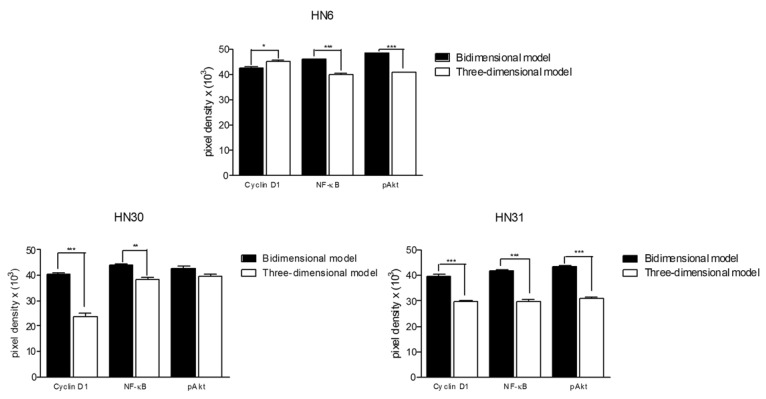
Western blot quantified bands (Pixels X Band area); Mean values plus standard deviation. Statistically significant differences are represented by * (p<0.05); ** (p<0.01); *** (p<0.001).

The comparison of protein levels between the experimental groups is presented in ( Table 1), (Fig. 2,3). The pAkt and NF-kB protein levels were reduced in the three-dimensional model. Cyclin D1 levels of HN30 and HN31 cell lines cultured three-dimensionally were decreased, but increased in the HN6 cell line.

**Table 1:**
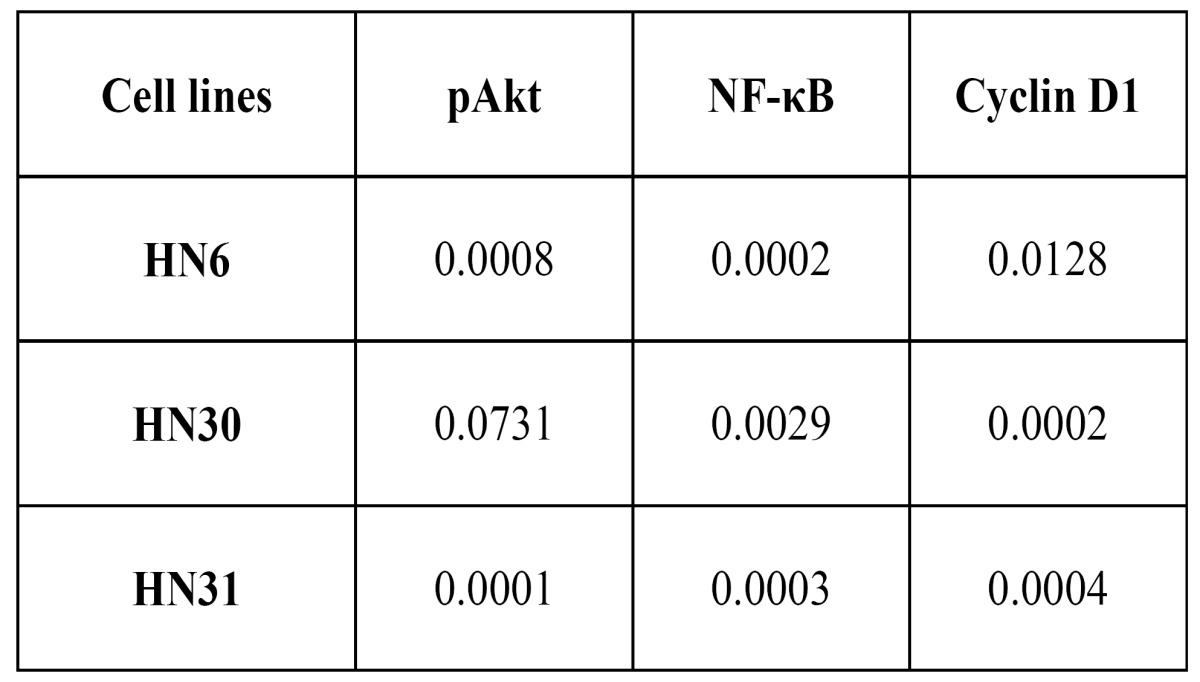
Respective p values obtained through the statistical comparison of bidimensional and three-dimensional Western blotting band quantifications.

## Discussion

The microenvironment plays an important role in the establishment and progression of carcinomas (15,16), and although bidimensional studies consist on a convenient and rapid method of analysis, the real recreation of in vivo microenvironments is not feasible by these means. Accordingly, three-dimensional cell culture models may reproduce the physiological context regarding cell morphology, adhesion, proliferation and migration (17).

Three-dimensional culture models are currently being used to understand the signals that regulate the normal function of tissues and how they may change during pathological processes, such as cancer (18). In fact, the tumor growth is dictated not only by the interaction between tumor cells, but also by the interaction of these cells with their surrounding stroma (19).

Recent studies have demonstrated that different culture models may induce changes in the cells behavior. In that way, matrix systems have lead to distinct results if compared to bidimensional substrates (20). One of these matrix systems, Matrigel, is a commercially obtained extracellular matrix that can provide microenvironmental support, mimicking the natural architecture of the cells in vivo (21). In this study, the three-dimensional culture was characterized by a marked colony growth pattern that was different from that found with the bidimensional culture model.

The Akt protein may be activated in both cytoplasm and the nucleus (22), and while the pAkt`s nuclear substrates are not yet totally described (23,24), cytoplasmatic pAkt leads to NF-κB activation (25). In this work, pAkt expression changed from the cytoplasm and nucleus to the nucleus solely, while the NF-κB and Cyclin D1 cellular localization showed no differences. Although NF-κB was not found inside the nuclei of the cells, nuclear transcription factors remain inside the nucleus only for a short period of time, indicating that the transcripting role of NF-κB in the HNSCC cells cannot be discarded (26).

All protein levels diminished in the three-dimensional model, excluding the Cyclin D1 levels in the HN6 cell line, which showed an increase. The change from a bidimensional to a three-dimensional model leads to a decrease in pAkt and NF-κB proteins levels, probably due to the cell adaptation period in Matrigel, which was characterized by the colony growth pattern (27) found herein. Additionally, Matrigel may influence cells behavior by reducing the stimulation of the PI3K-Akt signaling pathway. In that way, pAkt and NF-κB levels of HN6, HN30 and HN31 cell lines cultured with Matrigel were decreased.

On the other hand, the HN6 cell line has the EGFR (epidermal growth factor receptor) constitutively activated (14), and thus showed an increase on the Cyclin D1 levels. Despite that the adaptation period probably forced the reduction of pAkt and NF-κB levels, the EGFR activation of other signaling pathways as STAT3 and Ras/Map kinase (28) could be responsible for the Cyclin D1 increased levels in HN6 cells.

The present work showed interesting results about how the microenvironment may influence the behavior of carcinoma cells. The studied proteins, all of them related to carcinogenesis (8,9,28), presented reduced levels in the cells cultured with Matrigel, which demonstrates how this matrix system can imply in different results concerning tumor growth and invasiveness. Moreover, intrinsic characteristics, such as a constitutively activated EGFR, also influenced the cell behavior, regardless the microenvironment.

When working with three-dimensional matrix systems, a simple fact that may alter gene expression as well as cellular behavior is the density of the scaffold gel (29). Denser matrix may favor the proliferation of breast epithelial cells (30). In that way, the Matrigel concentration utilized in the present study was 1:3. Further studies may clarify if different gel concentrations could influence the behavior of HNSCCs cell lines.

Finally, the results encountered herein allowed us to conclude that the microenvironment influenced the HNSCC cells behavior by changing the expression of proteins related to tumor growth and cellular invasion. Additionally, intrinsic genetic conditions may have also played important roles upon the cells, despite the culture model employed.
